# Extensive Phenotypic Variability in Syndrome Dysmorphic Facies, Renal Agenesis, Ambiguous Genitalia, Microcephaly, Polydactyly, and Lissencephaly (DREAM-PL): A Case Report Highlighting Diagnostic and Management Challenges

**DOI:** 10.7759/cureus.54043

**Published:** 2024-02-12

**Authors:** Amin I Shaaban, Fikry M Lotfy, Mussaed S Alharbi, Ahmed F Zaky, Rand R Al Sari, Rakan K Mattar, Hussain A Al Mubarak, Amaal Jama, Shahad M Mousa, Nagham A Borah, Fatimah M Alshami, Futoon F Afandy, Sahar H Fadda

**Affiliations:** 1 Pediatrics, Saudi German Hospital, Jeddah, SAU; 2 Pediatrics, Batterjee Medical College, Jeddah, SAU

**Keywords:** variable expressivity, rare autosomal recessive disorder, neonatal case report, supportive care and symptom management, neonatal presentation, ctu2 gene mutation, dream-pl syndrome

## Abstract

The dysmorphic facies, renal agenesis, ambiguous genitalia, microcephaly, polydactyly, and lissencephaly (DREAM-PL) syndrome is a rare autosomal recessive disorder characterized by dysmorphic facies, renal agenesis, ambiguous genitalia in males, microcephaly, polydactyly, and lissencephaly. The CTU2 gene, which encodes a protein involved in the post-transcriptional modification of tRNAs is the source of the syndrome’s mutation. Several developmental abnormalities can result from a disruption of this modification, which is necessary for the proper translation of genes. The severity of the symptoms of DREAM-PL syndrome can range from moderate to severe, and its clinical characteristics are quite diverse. Some patients might have some of the distinguishing characteristics, whereas others might have all of them. The most typical characteristics include ambiguous genitalia, dysmorphic facies, and microcephaly. DREAM-PL syndrome is diagnosed based on clinical signs and genetic testing which can show mutations in the CTU2 gene. Although there is no known cure for this syndrome, the treatment aims to manage the symptoms. Other lines of treatment like surgical correction of birth defects can sometimes be beneficial to these patients in addition to supportive care. This study is a report of a 37-week-old male neonate, delivered by lower segment cesarean section. The baby’s birth weight is 2.760 kg with a heterozygous confirmed pathogenic mutation of the CTU2 gene confirmed by whole-exome sequencing.

## Introduction

CTU2 is one of the two cytosolic t-RNA thiouridylase proteins, the other being CTU1, which was initially identified in a genome-wide screen for "mutator" genes in *Caenorhabditis elegans* (*C. elegans*) [1]. Mutator genes get their name from the fact that their mutations produce genomic instability, which boosts mutagenesis [2]. One of the several posttranscriptional modifications of t-RNA that produce one or more changes that are thought to increase the accuracy and efficacy of protein translation is thiouridylation [3].

DREAM-PL syndrome is inherited in an autosomal recessive manner. The mutation that causes the syndrome can be traced back to the CTU2 gene [1]. The degree of DREAM-PL syndrome symptoms can range from mild to severe, and its clinical characteristics are highly variable. Some patients may have all the defining traits, while others may not [1]. CTU2 gene mutation causes variable brain, cardiac, and skeletal anomalies. The acronym DREAM-PL syndrome is mainly characterized by a combination of congenital anomalies which include dysmorphic facies, renal agenesis, ambiguous genitalia, microcephaly, polydactyly, and lissencephaly. There are other features seen in association with this gene that may present and lead to significant morbidity to the patient, including congenital heart defects such as atrial and ventricular septal defects, patent ductus arteriosus, and hypoplastic right ventricles, hypotonia, seizures, corpus callosum agenesis/dysgenesis, narrow forehead, depressed nasal bridge and hypoplastic maxilla, micrognathia, contractures of joints of the upper and lower extremities, bilateral talipes equinovarus, and micropenis [1].

The diagnosis is based on clinical symptoms and confirmed by genetic testing. A complete exome sequencing (ES) assay must be performed to detect CTU2 mutations.

Chromosomal microarray analysis (CMA) and ES are widely used for molecular genetics. diagnostics with variable diagnostic yields in highly consanguineous families or the presence of a family history of congenital anomalies [4]. Unfortunately, there is no definitive treatment available currently for DREAM-PL syndrome besides supportive treatment. Other lines of treatment like surgical correction of birth defects can sometimes be beneficial to these patients [1].

## Case presentation

**Figure 1:**
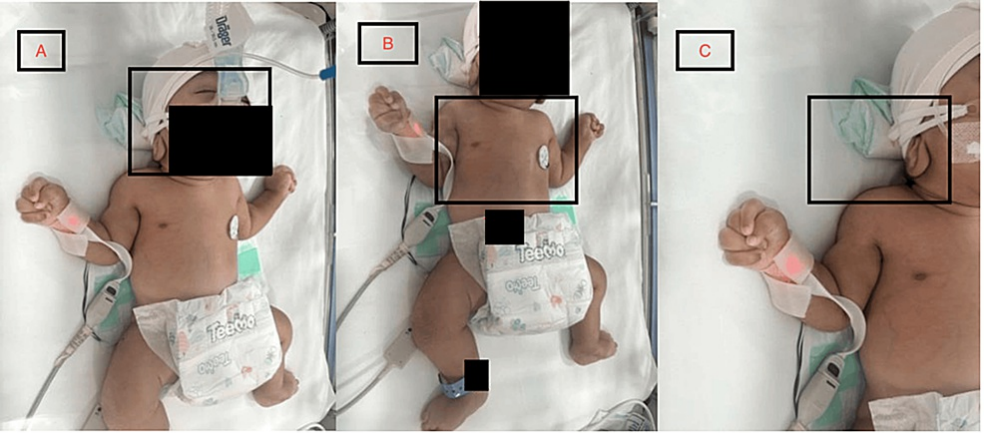
Physical examination of the patient A: Deep-seated small eyes and low-set large ears B: Short neck and wide-spaced nipples C: Large, right ear lobule

A 37-week-old male neonate was delivered by lower segment cesarean section. The baby’s birth weight is 2.760 kg with a 33 cm head circumference. On general examination, the patient was in bed, supine, oxygen-saturated, and maintained on ventilatory support. On head examination, the patient showed deep-seated small eyes, low-set large ears with large lobules, a high-arched palate, and micrognathia (Figure 1). Neck and chest examination showed a short neck and wide-spaced nipples (Figure 1).

**Figure 2:**
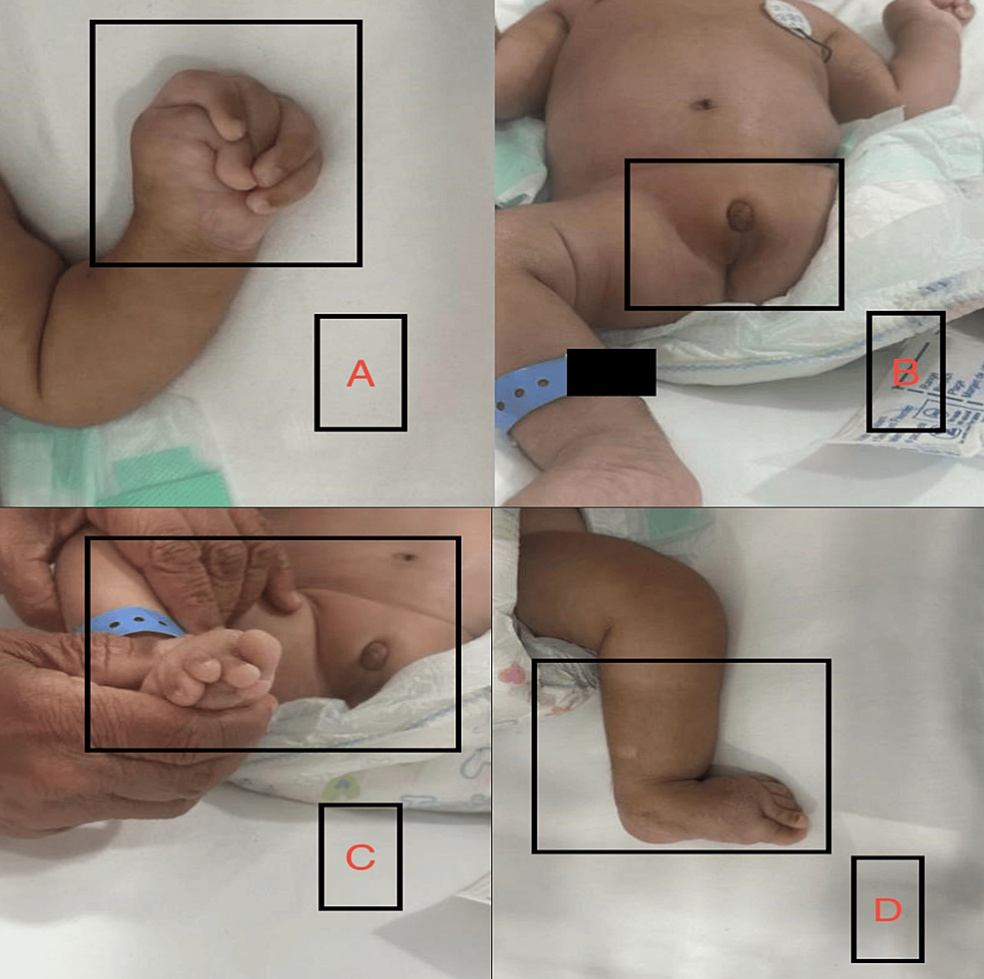
Physical examination of the patient A: Upper extremities overlapping of fingers of the left hand B: Ambiguous genitalia, a small penis, and a small empty scrotum C: Extremities overlapping of right foot toes D: General hypotonia and overlapping toes

An echocardiogram was made and it revealed a small atrial septal defect (ASD). Abdominal ultrasound showed a right renal mass. Abdominal examination was normal (Figure 2). The patient has ambiguous genitalia, a small penis, and a small empty scrotum (Figure 2). CNS examination revealed general hypotonia and microcephaly. The patient’s extremities show overcrowding of fingers of both hands (Figure 2).

Pelvic ultrasound revealed bilateral hip dislocation, bilateral talipes equinovarus, and developmental dysplasia of the hips (DDH). Whole-exome sequencing (WES) test was performed and the baby is a carrier (heterozygous) for a confirmed pathogenic mutation (CMP) of CTU2 (CTU2:NM_001012762.3:exon8:c.873G>A:p.Thr291) affecting splice site. Homozygous variants of CTU2 are frequently the cause of microcephaly, facial dysmorphism, renal agenesis, and ambiguous genitalia syndrome (autosomal recessive, OMIM#618142). The parents are first-degree cousins. WES test results for the parents were significant for having the same genetic mutation (CTU2) in both. Two previous children from the same family had similar features and died in the first year of life without performing genetic testing. The presented case is managed with respiratory support, spica for DDH and he is currently alive but is labeled as DNR (do-not-resuscitate) after confirming the diagnosis.

## Discussion

CTU2 is one of two t-RNA thiouridylase proteins found in the cytosol. It is originally discovered during a genome-wide search for "mutator" genes in *C. elegans* [5,6]. According to the literature, dysmorphic facies, renal agenesis, ambiguous genitalia in males, microcephaly, polydactyly, and lissencephaly are all congenital disorders related to CTU2 gene mutations. The term "DREAM- PL syndrome" refers to this clinical syndrome, which was initially brought up in 2016 [3].

Using the criteria of DREAM-PL to diagnose CTU2 gene mutations is potentially problematic, as only a fraction of the emphasized clinical features have been seen to present in the majority of the cases. In comparison to previous cases known to have DREAM-PL, although this patient exhibited most of the syndrome's characteristics except for lissencephaly, additional signs were also reported. Furthermore, there are other features seen in association with this gene that may present significant morbidity to a patient and are more likely to be observed by a provider early on affecting the overall clinical diagnosis. These include congenital heart defects and seizures, brain abnormalities such as corpus callosum hypoplasia, pituitary hypoplasia, and unspecified white matter loss. Therefore, according to this literature, using genetic testing is more accurate and specific than relying on DREAM-PL criteria [6].

Other literature has contrasting opinions and considers relying solely on genetic testing leads to underdiagnosed cases due to the limited diagnostic rate using genetic testing. They suggest improving the rapid genetic diagnosis in critically ill infants by combining rapid whole genome sequencing (rWGS) with novel algorithmic and diagnostic workflow: new software tools, RNA sequencing, long-read DNA sequencing, functional analysis studies, and yearly reanalysis [7]. Despite the benefit of having criteria to refer to clinically on prenatal ultrasounds, it is still recommended to use more specific prenatal molecular genetic diagnostics such as CMA and ES, especially in highly consanguineous families or in the presence of a family history of congenital anomalies [5].

Currently, there is no definitive treatment available for DREAM-PL syndrome besides supportive treatment as done in this case. However, there are some studies done on plants and fungi with a premise to guide future management [3,7]. The disease has a poor prognosis because of its multiorgan involvement and patients usually die within the first year of life.

## Conclusions

In summary, as seen in this patient, individuals with the CTU2 mutation present with a wide variation of clinical manifestations which may include but are not limited to dysmorphic facies, renal agenesis, ambiguous genitalia, microcephaly, polydactyly, and lissencephaly (DREAM-PL) syndrome. Thus, we suggest diagnosing this abnormality should mainly depend on molecular and genetic testing rather than the DREAM-PL criteria. Furthermore, due to the numerous fatal morbidities that the mutation presents with, the treatment of such cases is currently mostly symptomatic, and the majority of patients have a life expectancy of 1 year. For a better knowledge of the illness and its management options, further research and reporting is required.
